# Endocrine treatment near the end of life among older women with metastatic breast cancer: a nationwide cohort study

**DOI:** 10.3389/fonc.2023.1223563

**Published:** 2023-10-09

**Authors:** Máté Szilcz, Jonas W. Wastesson, Amaia Calderón-Larrañaga, Lucas Morin, Henrik Lindman, Kristina Johnell

**Affiliations:** ^1^ Department of Medical Epidemiology and Biostatistics, Karolinska Institutet, Stockholm, Sweden; ^2^ Aging Research Center, Department of Neurobiology, Care Sciences and Society, Karolinska Institutet & Stockholm University, Stockholm, Sweden; ^3^ Stockholm Gerontology Research Center, Stockholm, Sweden; ^4^ Inserm CIC 1431, University Hospital of Besançon, Besançon, France; ^5^ Inserm U1018, High-Dimensional Biostatistics for Drug Safety and Genomics, CESP, Villejuif, France; ^6^ Department of Immunology, Genetics and Pathology, Experimental and Clinical Oncology; Clinical Oncology, Faculty of Medicine, Uppsala University Hospital, Uppsala, Sweden

**Keywords:** metastatic breast cancer, end of life, endocrine treatment, palliative care, overtreatment, Sweden

## Abstract

**Background:**

The appropriate time to discontinue chemotherapy at the end of life has been widely discussed. In contrast, few studies have investigated the patterns of endocrine treatment near death. In this study, we aimed to investigate the end-of-life endocrine treatment patterns of older women with metastatic breast cancer and explore characteristics associated with treatment.

**Methods:**

A retrospective cohort study of all older women (age ≥65 years) with hormone receptor-positive breast cancer who died in Sweden, 2016 − 2020. We used routinely collected administrative and health data with national coverage. Treatment initiation was defined as dispensing during the last three months of life with a nine-month washout period, while continuation and discontinuation were assessed by previous use during the same period. We used log-binomial models to explore factors associated with the continuation and initiation of endocrine treatments.

**Results:**

We included 3098 deceased older women with hormone receptor-positive breast cancer (median age 78). Overall, endocrine treatment was continued by 39% and initiated by 5% and of women during their last three months of life, while 31% discontinued and 24% did not use endocrine treatment during their last year of life. Endocrine treatment continuation was more likely among older and less educated women, and among women who had multi-dose drug dispensing, chemotherapy, and CDK4/6 use. Only treatment-related factors were associated with treatment initiation.

**Conclusion:**

More than a third of women with metastatic breast cancer continue endocrine treatments potentially past the point of benefit, whereas late initiation is less frequent. Further research is warranted to determine whether our results reflect overtreatment at the end of life once patients’ preferences and survival prognosis are considered.

## Introduction

Breast cancer is the most common cancer among women worldwide (1). One-third of patients with metastatic breast cancer are 70 years or older at diagnosis (2). Many older patients (80%) have oestrogen- or progesterone receptor-positive tumours and are thereby candidates for endocrine treatment that blocks the hormone production or intervenes at the cell level of breast cancer (3). Patients may benefit from multiple lines of endocrine treatment (4), but there are no guidelines about how close to death endocrine treatment should be either continued, initiated or discontinued. At the end of life, many treatments’ potential adverse effects might outweigh their beneficial effects (e.g., chemotherapy), especially for older patients vulnerable to toxicities (5). Yet, little is known about endocrine treatment patterns in older women at the end of life.

A substantial body of research discusses the end-of-life trade-offs with anticancer therapies, especially chemotherapy (6–8). As the patients’ prognosis worsens, anticancer treatments often fail to comply with the general guiding principle that the “treatment should not be worse than the disease” due to the potential toxicities and negative impact on quality of life near death (9). Thus, chemotherapy is widely recognized as an overtreatment close to death (10, 11). Endocrine treatment is a less aggressive treatment option with easier parenteral administration and lower toxicity than chemotherapy for older individuals (12). However, endocrine treatment may increase the risk of cardiovascular events, osteoporosis, fatigue, pain, pneumonitis, neutropenia, hyperglycaemia, anxiety, depression, musculoskeletal complications, and other minor adverse events (13–16). These adverse effects are especially unwarranted in older frail people with multiple comorbidities near the end of life, when the goals of care should ideally shift toward comfort and quality-of-life-oriented care in line with patient preferences (17). Initiation of endocrine treatment in the last three months of life has been deemed inadequate, while continuing endocrine treatment until the end of life has been considered questionable in patients older than 75 years of age (18).

There are no comprehensive evaluations of endocrine therapy near death, mainly because only a few studies on end-of-life care include endocrine therapies in their definition of anticancer therapy (19–21). To fill these knowledge gaps, we first aimed to investigate the pattern of endocrine therapy in the last three months of life among older women with hormone receptor-positive metastatic breast cancer using routinely collected Swedish register data with national coverage. Secondly, we explored which patient characteristics were associated with endocrine therapy initiation versus no use, and continuation versus discontinuation at the end of life.

## Materials and methods

### Study design and population

This was a retrospective cohort study of decedents, that included all older women (age ≥65 years) with hormone receptor-positive metastatic breast cancer who died in Sweden between 1 January 2016 and 31 December 2020 (n = 5,045). We used routinely collected administrative and health data with national coverage in Sweden. Data from the National Cause of Death Register were linked using pseudonymised identifiers to the National Patient Register, the National Prescribed Drug Register, the Total Population Register, the Swedish Social Service Register and the Swedish Register of Education (**Supplementary eTable 1**).

**Figure 1:**
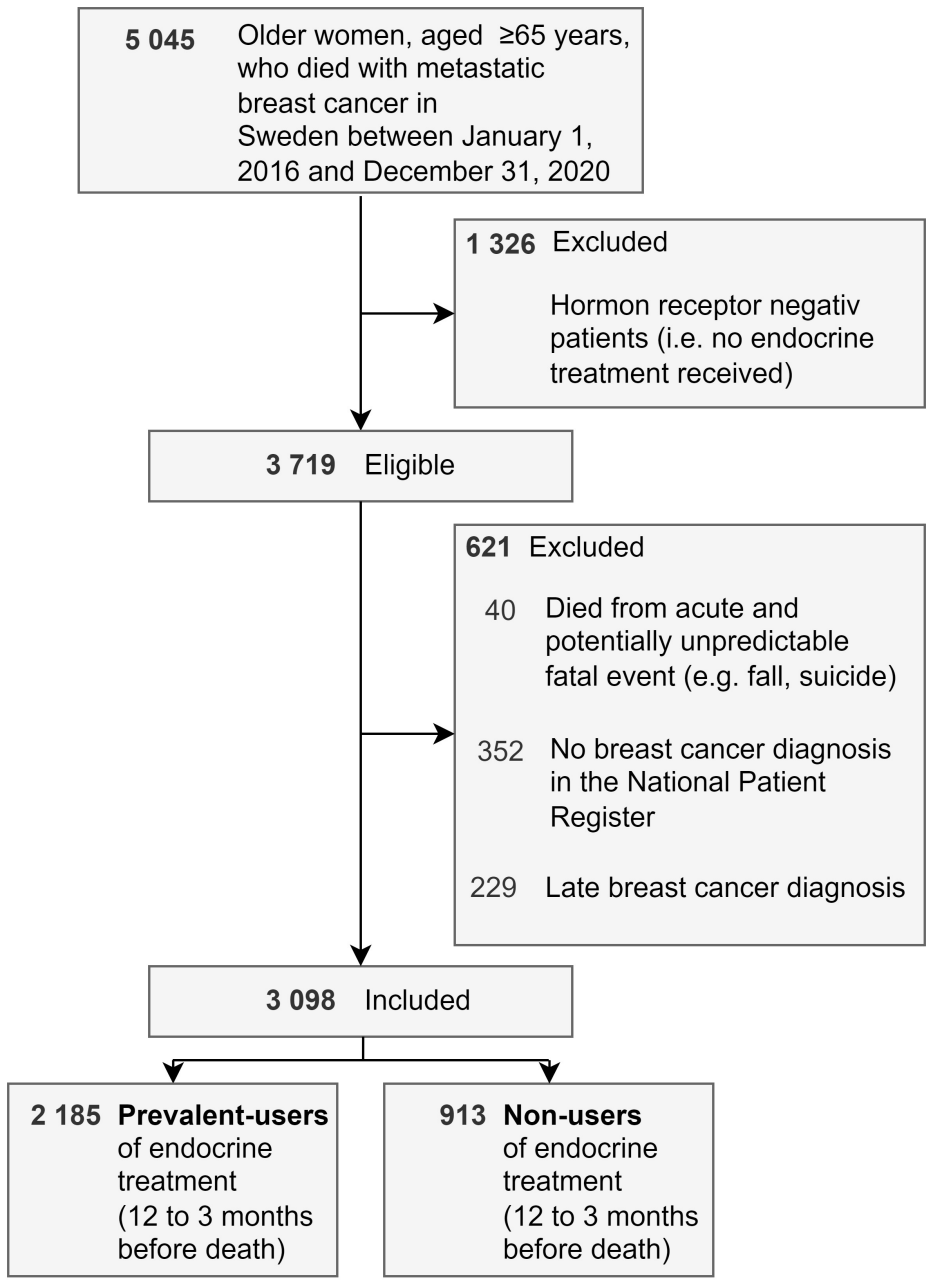
Flowchart.

Women were included in the cohort if they had both a diagnosis of breast cancer (International Statistical Classification of Diseases, 10th Revision [ICD-10] code C50) registered on the death certificate and if they had metastases (ICD-10: C78-79) registered in the National Patient Register or National Cause of Death Register (n=5,045) (**Supplementary eFigure 1**). Decedents were assumed to be hormone receptor positive, thus potentially eligible for endocrine treatment, if they had at least one endocrine treatment recorded in the National Prescribed Drug Register, similarly to previous research (22). Otherwise, they were excluded from the study. Decedents dying from a potentially acute and unpredictable fatal event according to their underlying cause of death (e.g., falls, suicide, stroke without history of ischemic heart disease) were excluded (n=40) from the analysis (**Supplementary eTable 2**). The rationale was to ensure that only women whose death may have potentially been anticipated by clinicians were included in the study. Furthermore, we excluded patients without breast cancer diagnosis registered in the National Patient Register during the last five years of life (n=352) and patients first diagnosed with breast cancer close to (≤3 months) death (n=229), because these patients might not have been regarded as end-of-life patients at the time of prescription. (**Figure 1**).

The study population was divided into prevalent users of endocrine treatment and non-users during the period ranging from twelve to three months before death. Those with at least one endocrine treatment prescription between twelve to three months before death were classified as prevalent users, while the remaining patients were classified as non-users. This division was needed to confirm which patients were “at risk” for continuing or initiating endocrine treatment.

### Outcome

Use of endocrine treatment was identified based on the Anatomical Therapeutic Chemical (ATC) codes (subgroup level ‘L02’ or everolimus [ATC: L01EG02]) from the National Prescribed Drug Register. Treatment exposure windows were constructed using a text parsing algorithm that calculates the prescribed daily dose based on the free text input of the prescriber. This method was described in detail elsewhere (23). We used four previously defined end-of-life treatment patterns (24): 1) *Treatment continuation* was defined as endocrine treatment dispensing during the last three months of life given previous use during twelve to three months before death; 2) *Treatment discontinuation* was defined as treatment dispensing during twelve to three months before death but no treatment dispensation during the last three months of life; 3) *Treatment initiation* was defined as the dispensing of endocrine treatment during the last three months of life, given a washout period of twelve to three months before death; and 4) *No use* was defined as no treatment dispensing during the last twelve months of life (**Supplementary eFigure 2**).

### Patient-level characteristics

We extracted sociodemographic characteristics of decedents (e.g., *sex*, *age at death*, *year of death*, *marital status*) from national registries. *Education* was defined as the lifetime highest attained educational level and was categorised into ‘primary/elementary’, ‘secondary’, and ‘tertiary’ education based on the ISCED-97 classification system (25). *Income quintiles* correspond to the latest individual disposable income obtained from Statistics Sweden’s longitudinal integrated database for health insurance and labour market studies. *Chronic multimorbidity* was operationalised as the number of chronic diseases out of a list of 60 pre-defined conditions (26), which were captured in the National Patient Register and National Prescribed Drug Register during the period ranging from five years to three months before death (**Supplementary eTable 3**).We calculated the *time since metastasis* diagnosis (‘<1 year’, ‘1-3 years’, ‘>3 years’) and the *Hospital Frailty Risk Score* (27) (‘low’, ‘moderate’, ‘high’) based on the same data (**Supplementary eTable 4**). The *cumulative time on endocrine treatment* was evaluated using the same timeframe (five years to three months before death) and categorised as ‘<1 year’, ‘1-2 years’, ‘>2 years’. *Nursing home residency* was considered permanent if registered at least once in the Swedish Social Service Register (28) between one year and three months before death. *Multi-dose drug dispensing* scheme [i.e., patients receive machine-dispensed drugs packed in disposable bags (29)] was identified between one year and three months before death using the Swedish Prescribed Drug Register. *Inpatient days* were the sum of hospitalised days during the three months before death and categorised as ‘1-14 days’, ‘15-30 days’, and ‘>30 days’. We also identified treatments during the last three months of life potentially administered alongside or instead of endocrine treatments: *cortisone* (ATC: H02AB), *CDK4/6* (palbociclib [ATC: L01EF01], ribociclib [ATC: L01EF02], abemaciclib [ATC: L01EF03]) or *hospital chemotherapy* (procedure codes [*in Swedish* KVÅ]: DT107, DT108, DT112, DT116, DT135) (30).

### Statistical analysis

For the primary analysis, we measured the proportion of decedents who continued, discontinued, initiated and did not use endocrine treatment during the last year of life to investigate the pattern of endocrine therapy use. For the secondary analysis, we used log-binomial generalised linear models. We explored mutually adjusted patient characteristics associated with a higher probability of endocrine therapy continuation compared to those who discontinued (reference category) in the prevalent-user cohort, and the associated factors with higher probability of initiation compared to constant no use (reference category) in the cohort of non-users. We chose log-binomial modelling strategy over logistic regression because the former outputs risk ratios (RR) that favour the interpretability of the results compared to the odds ratios generated by logistic regression. Additionally, odds ratios might overestimate the underlying risks in cohort studies when the outcome is common (31).

We performed three prespecified sensitivity analyses of our study’s primary aim where different cohort inclusion criteria were used. First, we included only patients aged 75 years or more because the Morin indicators (18), which categorised endocrine treatments as inadequate to initiate at the end of life were validated for this age group. Second, we included all the patients who died with breast cancer, regardless of metastatic status, because the ICD10 codes of C78 and C79 used to identify women with metastasis have not been validated in Sweden. However, these codes have previously been used to capture populations of metastatic breast cancer in Sweden (22, 32). Third, we included only those with breast cancer (ICD10-code: C50) as the underlying cause of death.

All analyses were performed with SAS software version 9.4 and R statistical software version 4.0.5.

### Guidelines and ethical approval

The present study was reported in keeping with the REporting of studies Conducted using Observational Routinely collected health Data (RECORD) guidelines (33) (**Supplementary eTable 5**) and was approved by the Regional Ethical Review Board of Stockholm (dnr: 2016/1001-31/4, 2020-03525; 2021-02004).

## Results

### Characteristics of the study population

We identified 5045 women aged 65 or more who died with metastatic breast cancer in Sweden between 1 January 2016 and 31 December 2020. Overall, 74% (n=3719) of individuals were assumed to be hormone receptor positive based on ever-receiving endocrine treatment prescription. We further excluded 33 patients who died from acute and potentially unpredictable fatal event, 352 patients who did not have any breast cancer diagnosis code registered in the National Patient Register, and 229 patients first diagnosed with breast cancer close (≤3 months) to death. The final study population consisted of 3098 decedents with a median age of 78 years (IQR 72–85) at death (**Table 1**). Large share (39%) of women had six or more comorbidities with a median of five (IQR 3–7) (**Supplementary eTable 7**). The prevalence of frailty was twelve per cent, and around eleven per cent lived in a nursing home. Patients spent a median of eleven days (IQR 1–23) in the hospital during the last three months of life. Almost half (48%) had accumulated more than two years of endocrine treatment prior to the last three months of life, and 28% of women were identified as a recipient of multi-dose drug dispensing. A small number of women (n=19, 0.6% of study population) died from COVID-19. The prevalent users who had endocrine treatment prescription between twelve to three months before death (n=2185; 71% of study population) were generally older, more frequently resided in a nursing home and used multi-dose drug dispensing scheme compared to the non-user group (n=913; 29% of study population).

**Table 1:** Characteristics of women who died with hormone receptor-positive metastatic breast cancer, aged ≥65 years in Sweden, 2016-2020.

Decedents, No.	Overall (n=3098)	Prevalent users 12 to 3 months before death (n= 2185)	Non-users 12 to 3 months before death (n=913)
Age at time of death			
Median (P_25_-P_75_), years	78.0 (72.0-85.0)	79.0 (72.0-86.0)	74.0 (70.0-81.0)
No. (%)			
65 to 74 years	1176 (38.0%)	716 (32.8%)	460 (50.4%)
75 to 84 years	1140 (36.8%)	817 (37.4%)	323 (35.4%)
85 years and older	782 (25.2%)	652 (29.8%)	130 (14.2%)
Education, No. (%)			
Primary/elementary	1089 (35.2%)	805 (36.8%)	284 (31.1%)
Secondary	1154 (37.2%)	807 (36.9%)	347 (38.0%)
Tertiary	812 (26.2%)	534 (24.4%)	278 (30.4%)
*Missing*	43 (1.4%)	39 (1.8%)	4 (0.4%)
Marital status			
Married	1178 (38.0%)	767 (35.1%)	411 (45.0%)
Single/divorced	878 (28.3%)	609 (27.9%)	269 (29.5%)
Widowed	1042 (33.6%)	809 (37.0%)	233 (25.5%)
Living arrangement			
Community-dwelling	2747 (88.7%)	1886 (86.3%)	861 (94.3%)
Nursing home	351 (11.3%)	299 (13.7%)	52 (5.7%)
Income quintiles			
Median (P25-P75), thousands SEK	1520 (1280-1890)	1520 (1290-1860)	1540 (1260-1960)
No. (%)			
Fifth (highest)	733 (23.7%)	488 (22.3%)	245 (26.8%)
Fourth	636 (20.5%)	462 (21.1%)	174 (19.1%)
Third	509 (16.4%)	386 (17.7%)	123 (13.5%)
Second	568 (18.3%)	404 (18.5%)	164 (18.0%)
First (lowest)	652 (21.0%)	445 (20.4%)	207 (22.7%)
Hospital Frailty risk score			
Median (P_25_-P_75_)	2.9 (0.8-6.4)	3.2 (0.9-6.6)	2.6 (0.6-5.8)
No. (%)			
Low (<5)	2050 (66.2%)	1418 (64.9%)	632 (69.2%)
Moderate (5–10)	684 (22.1%)	492 (22.5%)	192 (21.0%)
High (>10)	364 (11.7%)	275 (12.6%)	89 (9.7%)
Number of chronic diseases			
Median (P_25_-P_75_)	5.0 (3.0-7.0)	5.0 (3.0-7.0)	4.0 (3.0-6.0)
No. (%)			
0–1	305 (9.8%)	199 (9.1%)	106 (11.6%)
2–3	748 (24.1%)	513 (23.5%)	235 (25.7%)
4–5	845 (27.3%)	577 (26.4%)	268 (29.4%)
≥6	1200 (38.7%)	896 (41.0%)	304 (33.3%)
Years since metastasis			
Median (P25-P75)	1.7 (0.5-3.7)	1.4 (0.4-3.2)	2.7 (1.3-4.4)
No. (%)			
<1 year	1073 (34.6%)	901 (41.2%)	172 (18.8%)
1-3 years	1021 (33.0%)	694 (31.8%)	327 (35.8%)
>3 years	1004 (32.4%)	590 (27.0%)	414 (45.3%)
Multi-dose drug dispensing			
No	2232 (72.0%)	1470 (67.3%)	762 (83.5%)
Yes	866 (28.0%)	715 (32.7%)	151 (16.5%)
Years on endocrine treatment (last 5 years)			
Median (P25-P75)	1.9 (1.0-2.9)	2.0 (1.0-3.1)	1.6 (0.8-2.5)
No. (%)			
<1	787 (25.4%)	524 (24.0%)	263 (28.8%)
1 to 2	840 (27.1%)	548 (25.1%)	292 (32.0%)
>2	1471 (47.5%)	1113 (50.9%)	358 (39.2%)
Inpatient days in the last 3 months of life			
Median (P_25_-P_75_)	11.0 (1.0-23.0)	10.0 (0.0-22.0)	12.0 (3.0-25.0)
No. (%)			
No hospitalisation	757 (24.4%)	572 (26.2%)	185 (20.3%)
1–14 days	1077 (34.8%)	753 (34.5%)	324 (35.5%)
15–30 days	785 (25.3%)	542 (24.8%)	243 (26.6%)
≥30 days	479 (15.5%)	318 (14.6%)	161 (17.6%)
CDK4/6 use			
No	2979 (96.2%)	2098 (96.0%)	881 (96.5%)
Yes	119 (3.8%)	87 (4.0%)	32 (3.5%)
Hospital chemotherapy use			
No	2936 (94.8%)	2090 (95.7%)	846 (92.7%)
Yes	162 (5.2%)	95 (4.3%)	67 (7.3%)
Cortisone use			
No	1847 (59.6%)	1352 (61.9%)	495 (54.2%)
Yes	1251 (40.4%)	833 (38.1%)	418 (45.8%)

### End-of-life treatment patterns and associated factors

During the last three months of life, endocrine treatment was continued by 1,217 women with hormone receptor-positive metastatic breast cancer. This corresponds to 39% of the entire cohort and 56% of the prevalent users. In contrast, 968 patients discontinued treatment (31% of the entire cohort, 44% of prevalent users) during the last year of life. Endocrine treatment was initiated during the last three months of life by 157 women, which accounts for 5% of the cohort and 17% of the previously non-user groups. Overall, 756 women (24% of the entire cohort) did not receive endocrine treatment during the last year of life (**Table 2**).

**Table 2:** Endocrine treatment patterns at the end of life of women who died with hormone receptor-positive metastatic breast cancer, aged ≥65 years in Sweden, 2016-2020.

End-of-life treatment patterns	Overall (n=3098)	Prevalent users 12 to 3 months before death (n=2185)	Non-users 12 to 3 months before death (n=913)
Continuation	1217 (39.3%)	1217 (55.7%)	n/a
Discontinuation	968 (31.2%)	968 (44.4%)	n/a
Initiation	157 (5.1%)	n/a	157 (17.2%)
No use	756 (24.4%)	n/a	756 (82.8%)

n/a = not applicable.

Increased probability of endocrine treatment continuation (compared to discontinuation) was associated with higher age (RR_85+_ years: 1.26 [1.12-1.41]), multi-dose drug dispensing (RR: 1.22 [1.13-1.32]) and CDK4/6 use (RR 1.40 [1.25-1.58]) (**Table 3**). We found a lower probability of treatment continuation with higher education (RR_tertirary education_: 0.89 [0.81-0.98]) and chemotherapy use (RR: 0.66 [0.49-0.90]). Regarding treatment initiation (compared to no use), we found increased probability with the number of hospitalised days (RR_1-14 inpatient days_: 1.81 [1.12-2.91]), CDK4/6 use (3.16 [2.25-4.44]) and cortisone use (RR: 1.54 (1.17-2.04]). Those with earlier diagnosis of metastasis (RR_>3 years_: 0.49 [0.35-0.69]) or died in the year of 2020 (RR_2020_: 0.62 [0.38-1.00]) had lower propensity of treatment initiation.

**Table 3:** Relative risks estimates of factors associated with endocrine treatment continuation and initiation at the end of life of women who died with hormone receptor-positive metastatic breast cancer, aged ≥65 years in Sweden, 2016-2020.

	Continuation among prevalent users (N= 1217)			Initiation among previous non-users (N= 157)			
	%	RR (95% CI)	Adj. RR (95% CI)	%		RR (95% CI)	Adj. RR (95% CI)
Age at time of death, years							
65 to 74 years	44.0	1	1	18.0		1	1
75 to 84 years	56.2	1.29 (1.16-1.43)	1.17 (1.05-1.30)	15.5		0.86 (0.62-1.18)	0.99 (0.74-1.32)
85 years and older	67.9	1.57 (1.42-1.73)	1.26 (1.12-1.41)	18.5		0.98 (0.65-1.50)	1.26 (0.79-1.99)
Education, No. (%)							
Primary/elementary education	63.5	1	1	19.7		1	1
Secondary education	52.2	0.82 (0.76-0.89)	0.92 (0.86-1.00)	15.9		0.80 (0.57-1.13)	0.78 (0.56-1.09)
Tertiary education	48.7	0.77 (0.69-0.85)	0.89 (0.81-0.98)	16.2		0.82 (0.58-1.17)	0.80 (0.56-1.16)
Marital status							
Married	48.8	1	1	16.5		1	1
Single/divorced	52.1	1.06 (0.95-1.18)	1.03 (0.93-1.14)	18.6		1.10 (0.79-1.54)	1.19 (0.89-1.60)
Widowed	65.0	1.34 (1.23-1.46)	1.11 (1.01-1.22)	16.7		1.02 (0.71-1.46)	1.05 (0.71-1.55)
Living arrangement							
Community-dwelling	52	1	1	17.7		1	1
Nursing home	78.9	1.52 (1.41-1.64)	1.11 (1.01-1.22)	9.6		0.55 (0.23-1.27)	0.76 (0.32-1.79)
Income quintiles							
Fifth (highest)	52.5	1	1	14.7		1	1
Fourth	53.0	1.01 (0.90-1.14)	0.90 (0.82-1.00)	16.7		1.13 (0.72-1.78)	0.88 (0.59-1.33)
Third	60.6	1.15 (1.03-1.30)	0.95 (0.86-1.04)	13.8		0.89 (0.52-1.54)	0.63 (0.38-1.03)
Second	56.7	1.08 (0.96-1.22)	0.92 (0.83-1.03)	23.2		1.59 (1.05-2.39)	1.14 (0.77-1.69)
First (lowest)	56.9	1.08 (0.96-1.22)	0.96 (0.86-1.06)	17.9		1.23 (0.81-1.87)	0.91 (0.59-1.40)
Frailty							
Low (<5)	53.7	1	1	18.0		1	1
Moderate (5–10)	54.7	1.01 (0.92-1.11)	0.99 (0.91-1.08)	17.2		0.96 (0.68-1.37)	1.15 (0.79-1.67)
High (>10)	68.0	1.27 (1.16-1.40)	1.06 (0.97-1.15)	11.2		0.63 (0.35-1.16)	0.73 (0.39-1.39)
Number of chronic diseases							
0-1	51.3	1	1	20.8		1	1
2-3	55.8	1.09 (0.93-1.27)	1.06 (0.92-1.22)	17.9		0.86 (0.54-1.37)	0.89 (0.59-1.34)
4-5	53.2	1.03 (0.88-1.21)	1.01 (0.87-1.16)	16.4		0.78 (0.49-1.24)	0.82 (0.54-1.23)
>=6	58.3	1.15 (0.99-1.33)	1.01 (0.88-1.17)	16.1		0.78 (0.50-1.22)	0.82 (0.53-1.28)
Years since metastasis							
<1	60.2	1	1	30.2		1	1
1-3	53.0	0.89 (0.81-0.97)	0.96 (0.89-1.03)	16.8		0.57 (0.41-0.79)	0.62 (0.45-0.85)
>3	52.0	0.87 (0.79-0.95)	0.94 (0.86-1.02)	12.1		0.41 (0.29-0.57)	0.49 (0.35-0.69)
Multi-dose dispensing							
No	49.0	1	1	17.3		1	1
Yes	69.5	1.43 (1.33-1.54)	1.22 (1.13-1.32)	16.6		0.96 (0.65-1.42)	1.19 (0.84-1.68)
Years on endocrine treatment							
<1	56.7	1	1	24.7		1	1
1-2	55.5	0.97 (0.87-1.08)	1.01 (0.92-1.10)	13.7		0.56 (0.39-0.80)	0.62 (0.44-0.88)
>3	55.3	0.97 (0.89-1.07)	1.00 (0.92-1.08)	14.5		0.59 (0.43-0.82)	0.62 (0.45-0.85)
Inpatient days in the last 3 months of life							
No hospitalisation	64.7	1	1	10.8		1	1
1-14	53.9	0.82 (0.75-0.90)	0.93 (0.86-1.02)	20.4		1.89 (1.19-3.01)	1.81 (1.12-2.91)
15-30	54.6	0.84 (0.76-0.93)	0.97 (0.88-1.07)	19.3		1.80 (1.10-2.92)	1.50 (0.90-2.48)
>30	45.6	0.70 (0.61-0.80)	0.88 (0.77-1.01)	14.9		1.34 (0.76-2.34)	1.36 (0.77-2.38)
CDK4/6 use							
No	55.2	1	1	15.9		1	1
Yes	67.8	1.23 (1.05-1.43)	1.40 (1.25-1.58)	53.1		3.35 (2.34-4.8)	3.16 (2.25-4.44)
Hospital chemotherapy use							
No	56.8	1	1	17.4		1	1
Yes	30.5	0.54 (0.40-0.74)	0.66 (0.49-0.90)	14.9		0.86 (0.48-1.55)	0.85 (0.47-1.53)
Cortisone use							
No	58.9	1	1	14.7		1	1
Yes	50.5	0.86 (0.79-0.93)	0.96 (0.88-1.03)	20.1		1.37 (1.03-1.83)	1.54 (1.17-2.04)
Year of death							
2016	53.9	1	1	18.2		1	1
2017	56.0	1.06 (0.93-1.20)	1.04 (0.93-1.16)	19.0		1.08 (0.70-1.66)	1.09 (0.72-1.65)
2018	55.2	1.04 (0.92-1.18)	1.01 (0.91-1.13)	20.2		1.15 (0.74-1.77)	1.12 (0.74-1.70)
2019	58.1	1.09 (0.96-1.23)	1.08 (0.98-1.20)	19.4		1.09 (0.70-1.69)	0.98 (0.63-1.52)
2020	55.3	1.04 (0.92-1.17)	1.01 (0.91-1.12)	10.5		0.59 (0.36-0.98)	0.62 (0.38-1.00)

Percentages are calculated as a fraction of the patients continued or initiated treatment across the levels of the factors. Relative risks (RR) and 95% confidence intervals (CI) from log-binomial generalised linear models. Adjusted estimates are mutually adjusted. Further detailed results are presented in **Supplementary eTable 6**.

### Sensitivity analyses

The prespecified sensitivity analyses showed that endocrine treatment continuation was equally the highest (47%) when we included in the cohort only patients aged 75 years and older, or included all breast cancer patients regardless of metastatic status. The endocrine treatment continuation was the lowest (37%) across the cohorts when only patients with breast cancer (ICD10-code: C50) as the underlying cause of death were included. The proportions of endocrine treatment initiation was the lowest (4%) in the cohort aged 75 years and older. In all the sensitivity analyses, the proportions of patients discontinuing treatment were lower than in the primary cohort. (**Supplementary eTable 8**).

## Discussion

### Main findings

In this nationwide register-based-cohort study of women aged 65 years and older with hormone receptor-positive metastatic breast cancer, we found that endocrine treatment was initiated by 5% in the last three months of life and continued by more than one-third of the study population, potentially past the point of benefit. Initiation has been deemed often inadequate and continuation questionable at the end of life of older people (18). The large difference in initiating and continuing treatment suggests different clinical decision-making mechanisms at the end of life. In our explorative analysis, endocrine treatment continuation was associated with higher age, multi-dose drug dispensing scheme and CDK4/6 use, while we found a negative association for higher education and chemotherapy use. Treatment initiation had a positive association with more hospitalised days, CDK4/6 and cortisone use. Little attention has been given to endocrine treatment in breast cancer patients near death. The high prevalence of treatment continuation in the last three months of life should alert clinicians to re-evaluate patients’ treatments close to death and avoid treatment with limited or no benefits. Whether the extent of end-of-life treatment with systematic endocrine therapies is justified should be studied further, also weighing in patient’s preferences, performance status and survival prognosis.

### Interpretation and implications

The American Association of Clinical Oncologists suggests chemotherapy cessation near death (34), but no such recommendation is developed for systematic endocrine treatment at the end of life. The research on the appropriateness of end-of-life chemotherapy has possibly overshadowed discussions of other, less aggressive, systematic therapies such as endocrine treatments. Nevertheless, the potential adverse events of endocrine treatments (e.g., cardiovascular and musculoskeletal complications, depression) are non-negligible for older patients (13–16). Only a few studies include endocrine therapies in their definition of anticancer treatment (19–21). However, our estimates of older women who receive endocrine therapy near death surpass most estimates of the chemotherapy use (10, 11), indicating it should be considered when studying anticancer treatments. Overall, our findings are aligned with a study that reported a significant portion of women with breast cancer receive endocrine therapy alone or combined with chemo or immunotherapy at the end of life (21). Another study on patients with advanced cancer found that patients with breast cancer near death are at higher risk of receiving systematic anticancer therapy than other type of cancers due to the prevalent use of endocrine treatment (35).

We found that higher age is associated with the continuation (compared to discontinuation) of treatment. This contradicts earlier findings that older patients are less likely to receive anticancer treatment (36, 37), and that older age is associated with less likelihood of treatment continuation and initiation of prescribed drugs near death (24). Our results might partially reflect that patient involvement and shared decision–making decrease with age, resulting in continuation of routine prescriptions (38). We showed that higher educational attainment decreases the probability of end-of-life endocrine treatment continuation, which is supported by the notion that higher-educated individuals in general have higher health literacy and more control over their end-of-life care (39, 40). We found that chemotherapy use during the last three months was associated with lower a probability of endocrine treatment continuation, which might suggest a shift in disease management (i.e., attempt to relieve symptoms via palliative chemotherapy) (41). We further displayed that those who were not hospitalised are less likely to initiate treatment. This confirms the expectation that hospitalised patients receive more anticancer therapy at the end of life (20), which might be concerning given the increasing hospitalisation rates close to death (42). We showed a higher probability of endocrine treatment initiation and continuation if patients were also prescribed CDK4/6 treatment, which implies that they were in their earlier stages of the treatment course because CDK4/6 is recommended in first or second line (43). Furthermore, cortisone use was also associated with the initiation of endocrine treatment, indicating active adverse-effect control (e.g., headache, radicular pain) (41). Overall, our explorative findings should be considered hypothesis-generating, although they might also offer valuable insights to prescribers.

The considerable difference between the proportions of women initiating and continuing treatment at the end of life suggests a strong endowment effect (44): the aptitude not to initiate treatment but difficulty discontinuing. Furthermore, the continuation of endocrine treatment at a high rate at the end of life might be partially explained by the lower expected toxicity of endocrine treatments compared to other systematic anticancer therapies (e.g., chemotherapy, immunotherapy). Clinicians may view endocrine therapies as harmless and continue prescribing them, given their long-term indications. On the other hand, it might also reflect routine-based prescribing without carefully considering and re-evaluating the benefits and harms. Older people have fewer drug prescription changes, which is partly attributable to multi-dose drug dispensing (45). This is supported by our finding of increased probability of continuing treatment under the multi-dose drug dispensing scheme. Alternatively, our results might reflect that the women or their families want to continue these treatments regardless of potential adverse effects (46, 47). Patients and caregivers might equate continued treatment with not giving up, while clinicians might not want to deprescribe these medications to maintain hope (48, 49). Also, estimating the futility breaking point (i.e., the point in time after which the risks of the treatment outweigh its benefits) is both challenging and highly uncertain. However, simple tools like the ‘surprise question’ (50) or the Supportive and Palliative Care Indicators Tool (SPICT) (51) may help clinicians ascertain when treatment discontinuation should be discussed with the patient.

Our findings regarding women initiating endocrine treatment at the end of life might reflect several non-mutually exclusive mechanisms. Most importantly, estimating the remaining life expectancy of patients at the end of life is complicated (52). Clinicians may overestimate the benefit and underestimate the harms of the treatments because of cognitive bias (e.g., framing effects, impact and affect bias) (44, 53, 54). Also, patients might be considered well-functioning; thus, the active anticancer treatment seems appropriate. This might be reflected in our results, where we showed that those who had active treatment with CDK4/6 were also more likely to initiate endocrine treatment. While a study based on a panel of forty European experts classified endocrine treatments as inadequate to initiate at the end of life of older people (18), our study does not provide answers to whether the extent of treatment initiation we observed is indeed inadequate. However, our results highlight a further need to delineate potential pathways of treatment initiation of anticancer treatments at the end of life.

From a research perspective, our study highlights the need to further study to what extent systematic endocrine treatments can be considered as potential overtreatment. From a clinical practice perspective, we underline the importance of continuously re-evaluating patients’ ongoing prescriptions near death, especially among the older, less educated, and those with the multi-dose drug dispensing scheme. From a policy perspective, our study demonstrates a need to focus on quaternary prevention and advance care planning initiatives to prevent the overuse of medications at the end of life (55–57).

### Strengths and limitations

To our knowledge, this is the first Swedish study to evaluate endocrine treatment patterns at the end of life in a population with metastatic breast cancer. However, several limitations should be acknowledged. First, the mortality follow-back design potentially underestimates the prognostic uncertainty that the prescribers experienced (some women might have died unexpectedly, which could explain why endocrine treatments were initiated or continued near death). However, we attempted to mitigate this bias by excluding those who died from sudden and unexpected causes of death. Second, the clinical appropriateness of prescribing could not be determined based on administrative data alone, since, among other factors, patient preferences, functional status, disease severity and prognosis were unavailable (58); thus, the presented estimates should be interpreted with caution. Third, patients might have been misclassified as metastatic due to other primary cancers. However, all individuals in our study sample had a diagnosis of breast cancer in their death certificate as well as in their inpatient or outpatient records at least three months before death. We also performed several sensitivity analyses demonstrating that altering the study population does not meaningfully change the results. Fourth, the National Prescribed Drug Register only contains data about prescription drugs dispensed through pharmacies. Nursing home drug storerooms or hospital-dispensed drugs are not included, which might have led to an underestimation of endocrine treatment use. Also, it is impossible to ascertain from the register data whether the patient consumed the dispensed drug or not. Some patients may have stopped using the treatments earlier than our study results reflect. Fifth, patients were assumed to be hormone receptor-positive if they received an endocrine treatment prescription in the previous five years because immunohistochemistry test results are not recorded in the register. This is also why we did not further classify patients according to HER2-status. Sixth, the covariate of chemotherapy use might suffer from non-differential misclassification due to the potential underreporting of the procedure codes that were used to capture this variable (30). Seventh, we presented multiple estimates from the same model, which might introduce bias known as the “Table 2 fallacy” (59). Nonetheless, we regard this analysis as exploratory and hypothesis generating. Finally, our results may only be generalisable to settings similar to Sweden.

## Conclusion

In our nationwide cohort study, more than one third of older women with hormone receptor-positive metastatic breast cancer used endocrine treatments at the very last part of life, potentially past the point where treatment has a benefit. In addition, we found that endocrine treatment patterns differ across age, education, drug-dispensing scheme or among those with intensive treatments. Clinicians should be provided with training and resources to guide the deprescribing process near death of older individuals to reduce the burden of drug treatments and their potential harmful effects. Further research is warranted to determine whether our results reflect overtreatment at the end of life once patients’ preferences and survival prognosis are considered.

## Data availability statement

The data analyzed in this study is subject to the following licenses/restrictions: Data may be obtained from a third party and are not publicly available. Clinical data cannot be made publicly available because of privacy issues. However, additional results are available in the **Supplementary Files**. Requests to access these datasets should be directed to socialstyrelsen@socialstyrelsen.se.

## Ethics statement

The study was approved by the Regional Ethical Review Board in Stockholm (dnr: 2016/1001-31/4, 2020-03525, 2021-02004).

## Author contributions

MS, JW conceived and designed the study. MS performed the statistical analysis, interpreted the data, drafted, and critically revised the manuscript. JW, ACL, LM, HL and KJ interpreted the data and critically revised the manuscript. KJ obtained funding and acquired the data. KJ and JW provided supervision. KJ and MS are the guarantors of the study and data integrity. All authors contributed to the article and approved the submitted version.
