# Supplementary material for: Endocrine treatment near the end of life among older women with metastatic breast cancer: a nationwide cohort study
Source: Front Oncol. 2023 Oct 9;13:1223563. doi: 10.3389/fonc.2023.1223563 (PMC10591323; doi:10.3389/fonc.2023.1223563)
Supplement: Supplementary file 1 [file DataSheet_1.docx]

**SUPPLEMENTARY MATERIAL**

Table S1. A: Correlation matrix (Spearman coefficient) for the predictor variables. The correlation level is depicted by a progressive color gradient, ranging from green (inverse correlation) to red (direct correlation). B: Estimated R², tolerance (1 - R^2^) and Variance Inflation Factor (VIF) values related to correlation levels among predictor variables. Variables excluded for subsequent analysis are highlighted in bold. Mean sea level pressure, zonal and meridional winds, and sea surface temperature refer to anomaly trends (see text).

A

| Variables | Mean sea level  (mm·yr^-1^) | Zonal wind (m·s^-1^·yr^-1^) | Meridional wind  (m·s^-1^·yr^-1^) | Sea surface temperature (°C·yr^-1^) | Mean sea level pressure (Pa·yr^-1^) | Grain size (mm) | Tide range (m) | Beach slope | Dean's parameter | | Beach Width  Index | HMM 100 | HMM 500 | HMM 1000 |
| --- | --- | --- | --- | --- | --- | --- | --- | --- | --- | --- | --- | --- | --- | --- |
| Mean sea level (mm·yr^-1^) | 1 | 0.179 | 0.006 | 0.539 | -0.397 | -0.012 | 0.029 | -0.024 | | 0.079 | 0.116 | 0.086 | 0.087 | 0.087 |
| Zonal wind (m·s^-1^·yr^-1^) | 0.179 | 1 | 0.021 | 0.293 | -0.103 | 0.078 | 0.089 | 0.084 | | -0.139 | 0.038 | 0.134 | 0.133 | 0.138 |
| Meridional wind  (m·s^-1^·yr^-1^) | 0.006 | 0.021 | 1 | -0.116 | 0.312 | 0.059 | -0.278 | 0.150 | | 0.063 | -0.194 | -0.015 | -0.015 | 0.003 |
| Sea surface temperature (°C·yr^-1^) | 0.539 | 0.293 | -0.116 | 1 | -0.416 | -0.010 | 0.230 | -0.009 | | 0.113 | 0.173 | 0.283 | 0.285 | 0.315 |
| Mean sea level pressure (Pa·yr^-1^) | -0.397 | -0.103 | 0.312 | -0.416 | 1 | 0.130 | -0.252 | -0.030 | | -0.035 | -0.198 | -0.162 | -0.161 | -0.176 |
| Grain size (mm) | -0.012 | 0.078 | 0.059 | -0.010 | 0.130 | 1 | -0.135 | 0.694 | | -0.503 | -0.227 | 0.041 | 0.039 | 0.041 |
| Tide range (m) | 0.029 | 0.089 | -0.278 | 0.230 | -0.252 | -0.135 | 1 | -0.341 | | 0.096 | 0.743 | 0.165 | 0.168 | 0.186 |
| Beach slope | -0.024 | 0.084 | 0.150 | -0.009 | -0.030 | 0.694 | -0.341 | 1 | | -0.505 | -0.445 | 0.013 | 0.010 | 0.023 |
| Dean's parameter | 0.079 | -0.139 | 0.063 | 0.113 | -0.035 | -0.503 | 0.096 | -0.505 | | 1 | 0.224 | -0.129 | -0.129 | -0.116 |
| Beach Width Index | 0.116 | 0.038 | -0.194 | 0.173 | -0.198 | -0.227 | 0.743 | -0.445 | | 0.224 | 1 | 0.079 | 0.081 | 0.085 |
| HMM 100 | 0.086 | 0.134 | -0.015 | 0.283 | -0.162 | 0.041 | 0.165 | 0.013 | | -0.129 | 0.079 | 1 | 0.999 | 0.953 |
| HMM 500 | 0.087 | 0.133 | -0.015 | 0.285 | -0.161 | 0.039 | 0.168 | 0.010 | | -0.129 | 0.081 | 0.999 | 1 | 0.954 |
| HMM 1000 | 0.087 | 0.138 | 0.003 | 0.315 | -0.176 | 0.041 | 0.186 | 0.023 | | -0.116 | 0.085 | 0.953 | 0.954 | 1 |

B

| Statistics | Mean sea level  (mm·yr^-1^) | Zonal wind  ( m·s^-1^·yr^-1^) | Meridional wind  ( m·s^-1^·yr^-1^) | Sea surface temperature  (°C·yr^-1^) | Mean sea level pressure  (Pa·yr^-1^) | Grain size  (mm) | Tide range  (m) | Beach slope | Dean's parameter | Beach Width Index | **HMM 100** | **HMM 500** | HMM 1000 |
| --- | --- | --- | --- | --- | --- | --- | --- | --- | --- | --- | --- | --- | --- |
| R² | 0.407 | 0.127 | 0.227 | 0.468 | 0.413 | 0.569 | 0.618 | 0.647 | 0.379 | 0.609 | **0.998** | **0.998** | 0.515 |
| Tolerance | 0.593 | 0.873 | 0.773 | 0.532 | 0.587 | 0.431 | 0.382 | 0.353 | 0.621 | 0.391 | **0.002** | **0.002** | 0.485 |
| VIF | 1.687 | 1.146 | 1.294 | 1.878 | 1.704 | 2.322 | 2.620 | 2.837 | 1.611 | 2.560 | **587.327** | **594.315** | 4.724 |

Table S2: Correlation matrix among predictor variables, calculated after removing HMM100 and HMM500 as highly correlated variables. The colors indicate the level of correlation between each pair of variables using an ascending gradient from white to red. Mean sea level pressure, zonal and meridional winds, and sea surface temperature refer to anomaly trends (see text).

|  | Mean sea level  (mm·yr^-1^) | Zonal wind  (m·s^-1^yr^-1^) | Meridional wind  (m·s^-1^·yr^-1^) | Sea surface temperature  (°C·yr^-1^) | Mean sea level pressure  (Pa·yr^-1^) | Grain size  (mm) | Tide range  (m) | Beach slope | Dean's parameter | Beach Width Index | HMM 1000 |
| --- | --- | --- | --- | --- | --- | --- | --- | --- | --- | --- | --- |
| Mean sea level (mm·yr^-1^) | **1** | 0.171 | -0.011 | 0.529 | -0.397 | 0.019 | 0.004 | 0.013 | 0.057 | 0.098 | 0.102 |
| Zonal wind (m·s^-1^·yr^-1^) | 0.171 | **1** | 0.034 | 0.296 | -0.109 | 0.073 | 0.085 | 0.08 | -0.123 | 0.034 | 0.134 |
| Meridional wind (m·s^-1^·yr^-1^) | -0.011 | 0.034 | **1** | -0.099 | 0.316 | 0.043 | -0.291 | 0.131 | 0.082 | -0.212 | -0.01 |
| Sea surface temperature (°C·yr^-1^) | 0.529 | 0.296 | -0.099 | **1** | -0.415 | -0.03 | 0.211 | -0.029 | 0.12 | 0.168 | 0.317 |
| Mean sea level pressure  (Pa·yr^-1^) | -0.397 | -0.109 | 0.316 | -0.415 | **1** | 0.132 | -0.242 | -0.02 | -0.061 | -0.196 | -0.182 |
| Grain size (mm) | 0.019 | 0.073 | 0.043 | -0.03 | 0.132 | **1** | -0.123 | 0.687 | -0.502 | -0.225 | 0.048 |
| Tide range (m) | 0.004 | 0.085 | -0.291 | 0.211 | -0.242 | -0.123 | **1** | -0.345 | 0.079 | 0.746 | 0.182 |
| Beach slope | 0.013 | 0.08 | 0.131 | -0.029 | -0.02 | 0.687 | -0.345 | **1** | -0.501 | -0.448 | 0.011 |
| Dean’s parameter | 0.057 | -0.123 | 0.082 | 0.12 | -0.061 | -0.502 | 0.079 | -0.501 | **1** | 0.21 | -0.093 |
|  | 0.098 | 0.034 | -0.212 | 0.168 | -0.196 | -0.225 | 0.746 | -0.448 | 0.21 | **1** | 0.092 |
| Beach Width Index |  |  |  |  |  |  |  |  |  |  |  |
| HMM 1000 | 0.102 | 0.134 | -0.01 | 0.317 | -0.182 | 0.048 | 0.182 | 0.011 | -0.093 | 0.092 | **1** |

Table S3: Values for all variables of the central objects within each cluster. The central object of a cluster refers to the element within the cluster that exhibits the closest proximity to the cluster centroid. The central objects are defined by beaches whose characteristics closely resemble those of the estimated centroids. Mean sea level pressure, zonal and meridional winds, and sea surface temperature refer to anomaly trends (see text).

| Cluster (central object) | Mean sea level  (mm·yr^-1^) | Zonal wind  (m·s^-1^·yr^-1^) | Meridional wind  (m·s^-1^·yr^-1^) | Sea surface temperature  (C°·yr^-1^) | Mean sea level pressure  (Pa·yr^-1^) | Grain size  (mm) | Tide range  (m) | Beach slope | Dean's parameter | Beach Width index | HMM 1000 |
| --- | --- | --- | --- | --- | --- | --- | --- | --- | --- | --- | --- |
| 1 (Grande SFS) | 1.717 | -0.003 | 0.017 | 0.020 | 0.303 | 0.440 | 2.000 | 0.111 | 3.320 | 18.018 | 0.159 |
| 2 (Louro) | 0.440 | -0.002 | -0.004 | 0.014 | 1.329 | 0.350 | 3.200 | 0.053 | 2.850 | 60.377 | 0.460 |
| 3 (Majorlandia) | 2.294 | -0.010 | 0.035 | 0.014 | 1.424 | 0.250 | 3.300 | 0.022 | 4.700 | 150.150 | 0.557 |
| 4 (Campwin) | 2.117 | -0.009 | -0.005 | 0.014 | -1.195 | 0.270 | 6.500 | 0.022 | 2.780 | 295.455 | 0.416 |
| 5 (Armstrongs) | 2.747 | -0.009 | -0.004 | 0.014 | -1.210 | 0.220 | 6.500 | 0.009 | 3.700 | 722.222 | 0.276 |


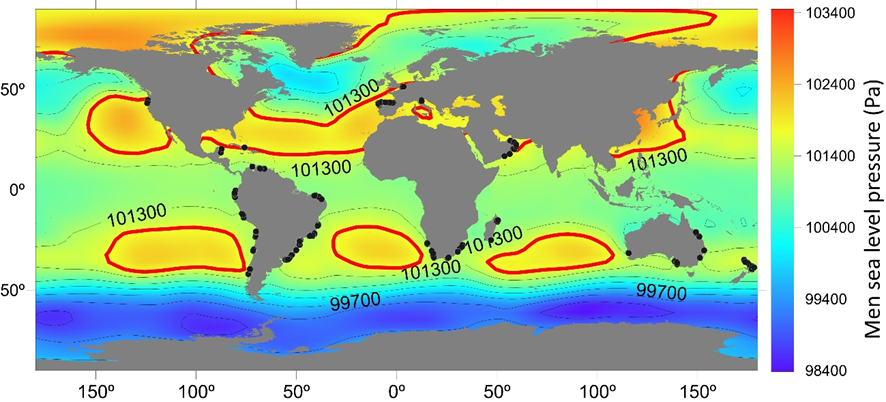


Figure S1. Global long-term average (1982 -2021) of mean sea level pressure highlighting the position of high-pressure centers (red contours), generally at lower latitudes than the mean sea level pressure anomalies shown in Figure 1. For example, southern high-pressure centers are roughly located at 32°S, while the high-pressure anomalies are over 40°S.


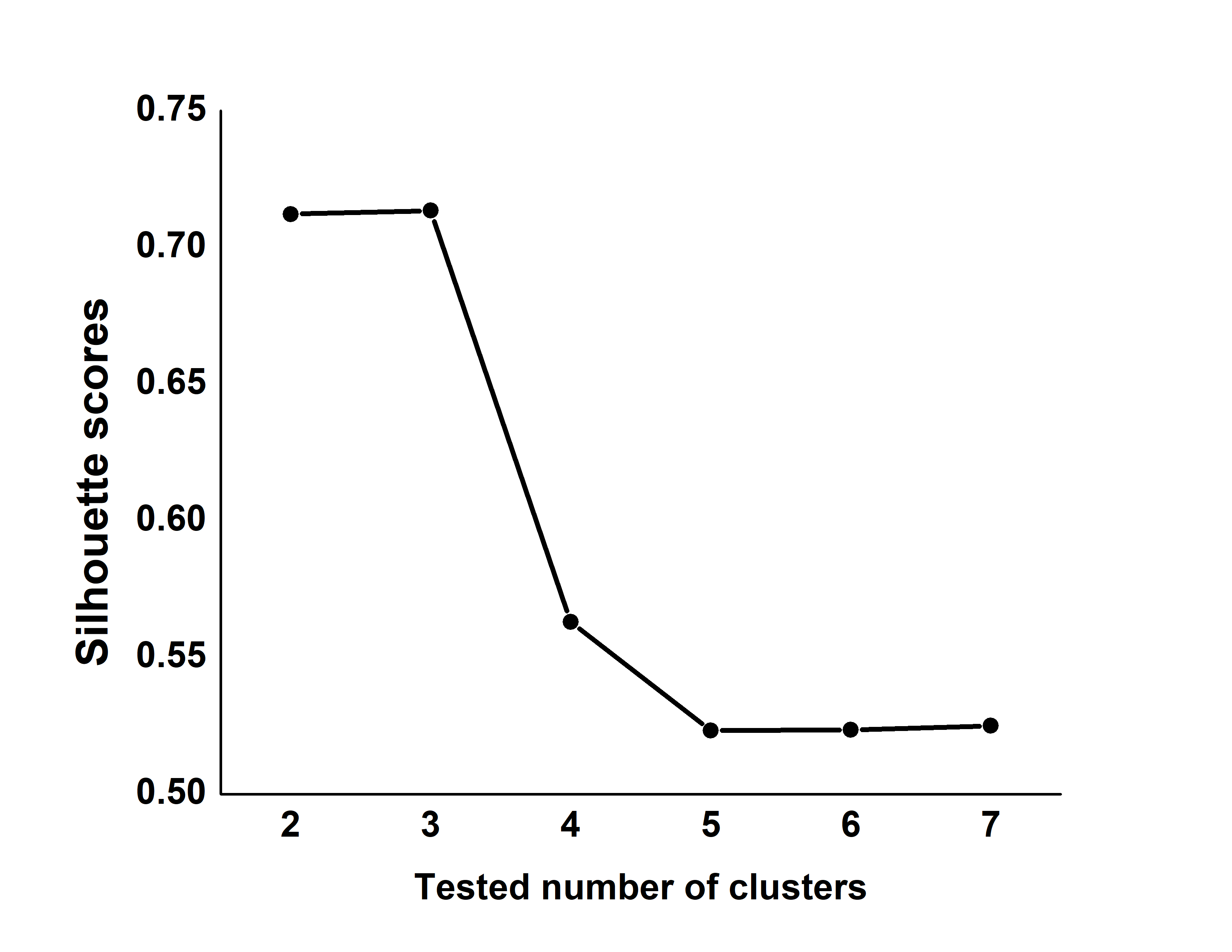


Figure S2. Trend of the Silhouette score for the tested number of clusters (2-7) using the k-means algorithm in the global beach database comprising 315 sandy beaches from five continents. The estimated score for 5 clusters was > 0.50, indicating a significant degree of separation between clusters and therefore meaningful and reliable clustering results (Figure S1).


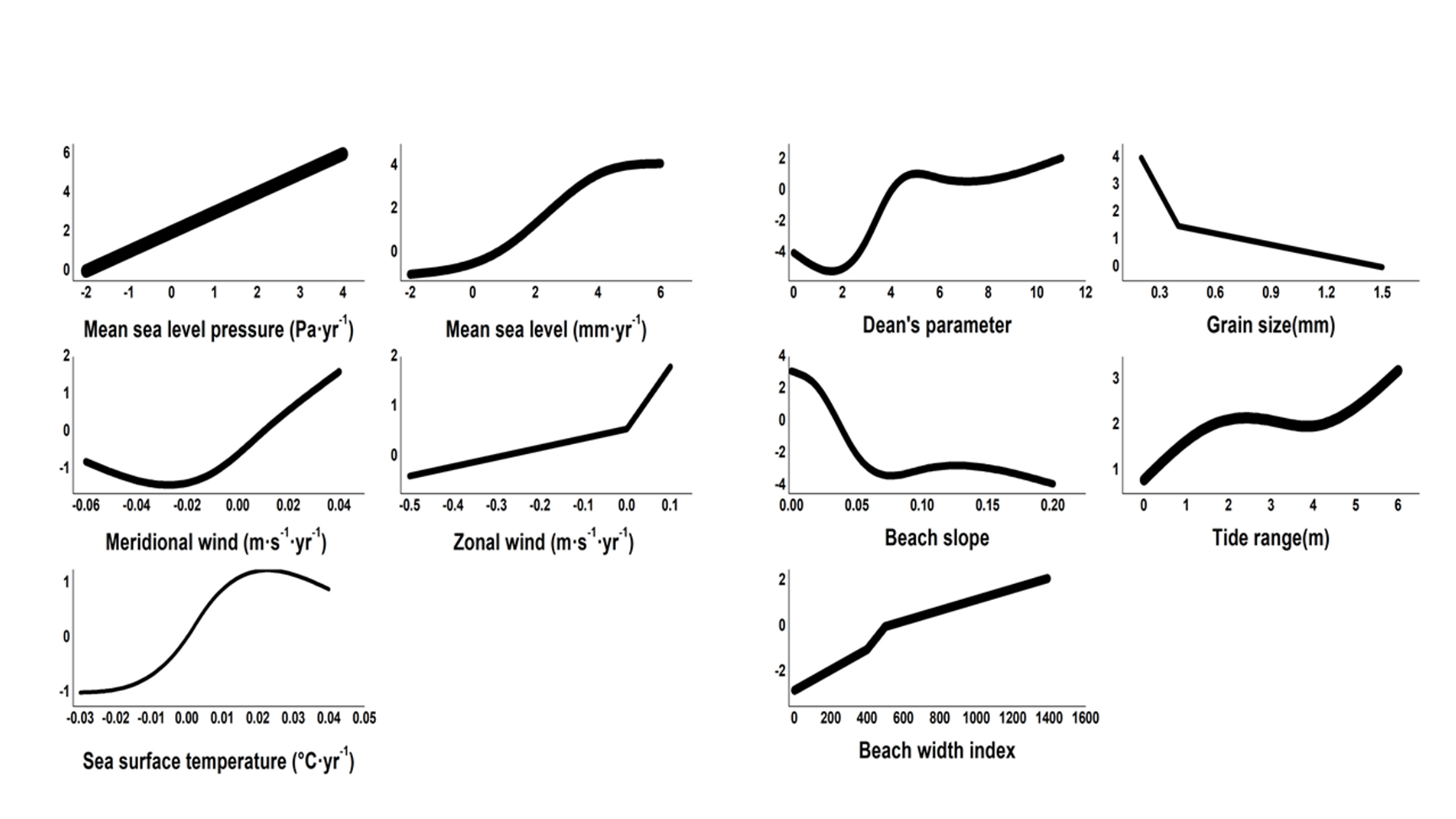


Figure S3. Partial dependence plots showing the associations between erosion rates and local and regional predictor variables. Line thickness is proportional to the importance of each variable, determined using random forest analyses (see Figure 6) and calculated separately for each response variable through Out-Of-Bag error (OOB) estimation. Note the different Y-axis scales. Mean sea level pressure, zonal and meridional winds, and sea surface temperature refer to anomaly trends (see text).


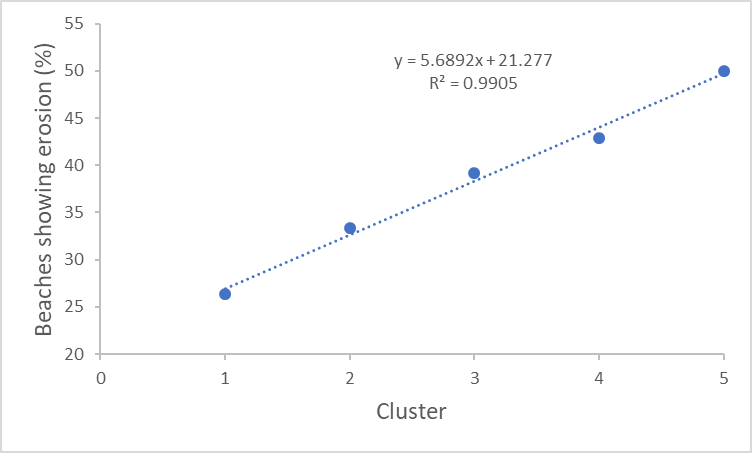


Figure S4. Relative representation of beaches experiencing erosion rates, discriminated by cluster identified using the k-means algorithm (see text for details).
